# Different proxies, different stories? Imperfect correlations and different determinants of fitness in bighorn sheep

**DOI:** 10.1002/ece3.9582

**Published:** 2022-12-08

**Authors:** Joanie Van de Walle, Benjamin Larue, Gabriel Pigeon, Fanie Pelletier

**Affiliations:** ^1^ Biology Department Woods Hole Oceanographic Institution Woods Hole Massachusetts USA; ^2^ Département de Biologie Université de Sherbrooke Sherbrooke Québec Canada; ^3^ Institut de recherche sur les forêts Université du Québec en Abitibi‐Témiscamingue Rouyn‐Noranda Québec Canada

**Keywords:** bighorn sheep, correlation, gradient boosting, individual fitness, lifetime reproductive success, selection

## Abstract

Measuring individual fitness empirically is required to assess selective pressures and predicts evolutionary changes in nature. There is, however, little consensus on how fitness should be empirically estimated. As fitness proxies vary in their underlying assumptions, their relative sensitivity to individual, environmental, and demographic factors may also vary. Here, using a long‐term study, we aimed at identifying the determinants of individual fitness in bighorn sheep (*Ovis canadensis*) using seven fitness proxies. Specifically, we compared four‐lifetime fitness proxies: lifetime breeding success, lifetime reproductive success, individual growth rate, individual contribution to population growth, and three multi‐generational proxies: number of granddaughters, individual descendance in the next generation, and relative genetic contribution to the next generation. We found that all proxies were positively correlated, but the magnitude of the correlations varied substantially. Longevity was the main determinant of most fitness proxies. Individual fitness calculated over more than one generation was also affected by population density and growth rate. Because they are affected by contrasting factors, our study suggests that different fitness proxies should not be used interchangeably as they may convey different information about selective pressures and lead to divergent evolutionary predictions. Uncovering the mechanisms underlying variation in individual fitness and improving our ability to predict evolutionary change might require the use of several, rather than one, the proxy of individual fitness.

## Graphical abstract

Fitness is central to ecology and evolution, but it is typically proxied using several contrasting approaches, which can lead to contrasting conclusions about fitness determinants and selection. Using seven proxies of individual fitness in bighorn sheep, we showed that fitness proxies were positively correlated, but the magnitude of the correlations varied substantially. We also showed that depending on the fitness proxy, fitness determinants and predictions about selection varied, suggesting that fitness proxies should not be used interchangeably.

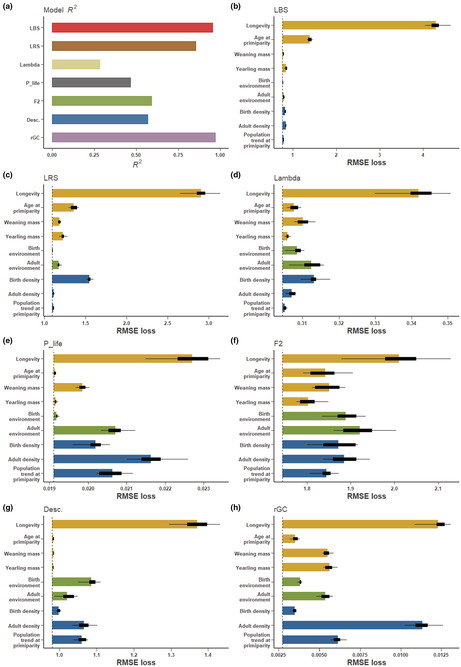

## 1 INTRODUCTION

From Darwin's seminal book in 1859 (Darwin, 1859) to our contemporary conception of evolution, it is unequivocal that for heritable phenotypic traits to evolve, these traits must affect the fitness of individuals expressing them (Freeman & Herron, 2007). However, individual fitness in ecology lacks an unambiguous practical definition and has been the source of much semantic confusion and debate (Ariew & Lewontin, 2004; Kokko, 2021; Matthen & Ariew, 2002; Orr, 2009; Roff, 2008). Darwin referred to individuals that were best able to survive and reproduce in their environment as being “fitter” than others. Darwin, however, did not provide a clear, measurable, definition of this quantity, leaving it up to interpretation. Since Darwin's pioneering work, the concept of fitness has branched into numerous nuanced definitions (Ariew & Lewontin, 2004; Matthen & Ariew, 2002). Nevertheless, most modern definitions share the common ground that fitness should reflect the expected representation of an individual's genes in a population at a certain time in the future (Orr, 2009; Roff, 2008; Sæther & Engen, 2015; Stearns, 1992). It is, crucially, individual differences in genetic contributions to future generations that generate evolutionary adaptation through selection (Falconer & Mackay, 1996; Lande & Arnold, 1983; Roff, 1993).

To measure the strength of selection and make predictions about evolutionary changes in wild populations, individual phenotypes are typically regressed against individual fitness (Falconer & Mackay, 1996). However, individual fitness is sensitive to changes in demographic, environmental, and social contexts under which individuals live and reproduce (Dobson et al., 2020; Lardy et al., 2015; Metcalf & Pavard, 2007; Pigeon & Pelletier, 2018). For instance, early‐life conditions can have long‐lasting consequences on the reproductive performance of entire cohorts (Albon et al., 1987; Pigeon & Pelletier, 2018). Interactions between the timing of reproduction and population dynamics can also yield different fitness outcomes, with early reproduction favored in growing or stable populations and late reproduction in declining populations (Brommer et al., 2002; Mcgraw & Caswell, 1996). Measuring selection and ultimately making inferences about evolutionary changes in the wild thus requires a good understanding of the factors influencing individual fitness, including individual traits, environmental fluctuations, and density‐dependence (reviewed in Sæther & Engen, 2015).

Unfortunately, an important complication arises as the conceptual confusion around fitness inevitably translates to confusion around its estimation in practice. Estimating fitness using empirical data can be further complicated by limitations in the monitoring of wild individuals, such as incomplete parental assignation, and difficulty in tracking descendance. As depicted in Ariew and Lewontin (2004), fitness appears incommensurable as there is no single measure used by empiricists. Lifetime Reproductive Success (LRS), or the total number of offspring produced over an individual's lifetime, is likely the simplest, most convenient, and most frequently used fitness proxy in vertebrate studies (Clutton‐Brock, 1988; Gustafsson, 1986; Hayward et al., 2014; Le Boeuf et al., 2019). LRS is sometimes measured as the number of offspring conceived, born, or hatched over the lifespan of an individual (Hayward et al., 2014; Lukas & Clutton‐Brock, 2014). However, when juvenile mortality is high and nonrandom (Martin et al., 2018; McAdam et al., 2007; Potti et al., 2013), considering offspring at later life stages, such as at fledging in birds or weaning in mammals, has been suggested to better reflect longer‐term genetic contributions of parents (Le Boeuf et al., 2019; McLoughlin et al., 2006). However, this approach raises the question of “whose fitness is it?” (Wolf & Wade, 2001) and blurs the line between the response to selection and selection itself (Wilson et al., 2005). Because the life stage at which offspring are considered varies across studies, comparisons of LRS and its determinants in different contexts are often impractical (Brommer et al., 2002).

Many studies have underlined that LRS does not reflect demographic context and timing of reproductive events over the lifetime (Caswell, 2001; Mcgraw & Caswell, 1996; Reid et al., 2019). Some have argued that because fitness is inseparable from demography, fitness should be considered in the light of evolutionary demography (Metcalf & Pavard, 2007; Pelletier et al., 2007). Indeed, individual performance, like population growth, can be seen as the cumulative contribution of reproductive and survival rates. Mcgraw and Caswell (1996) derived a demography‐related proxy of fitness, termed individual growth rate (λ_i_). This measure, analogous to the population‐wide intrinsic rate of increase, combines information on age‐specific individual survival and reproductive rates to yield an approximation of the rate at which a population consisting of only the focal individual would be expected to increase in size over time. This approach is rate‐sensitive (Brommer et al., 2002), and can directly incorporate information on the timing of reproductions to account for the relative benefits of reproducing at different ages under different contexts of population growth (Houston & McNamara, 1999). Other methods advocate that fitness should be proxied relative to other individuals in the population with which they compete and population size instead of being measured as absolute values (Coulson et al., 2006; Wilson, 2004). One such proxy calculates the sum of annual contributions to population growth of each focal individual relative to other individuals in the population each year during the lifetime (P_life; Coulson et al., 2006; Watson & Galton, 1875). Finally, since the “fittest” individuals should yield the largest genetic contributions to future generations, some consider estimates of genetic contributions over more than one generation in the future as ultimate fitness proxies (Brommer et al., 2002). However, sufficiently detailed and deep enough pedigrees to estimate long‐term contributions can be obtained in human populations (Bherer et al., 2011) but are still rare in wild vertebrate populations (Reid et al., 2019).

Considering the importance of individual fitness in evolutionary studies, several recent studies on short‐lived vertebrate species have assessed how different fitness proxies compare with one another (Alif et al., 2022; Viblanc et al., 2022). However, estimating fitness over an individual's lifetime and over more than one generation is much more challenging in long‐lived species. Moreover, little is known about how the relative contribution of individual, environmental and demographic factors varies according to which fitness proxy is used. Here, using more than 25 years of longitudinal data on female bighorn sheep (*Ovis canadensis*), a long‐lived species, our objective was twofold: (1) measure and compare proxies of fitness that can be calculated in wild vertebrate populations and (2) quantify the relative importance of individual characteristics, environmental conditions, and demography in explaining variation in each fitness proxy to assess whether the determinants of fitness differ according to the proxy used. Some fitness proxies we considered are commonly used in vertebrate studies, but we also considered new or infrequently used proxies, such as the relative genetic contribution to the next generation, which has been developed and applied in the context of human demography (Bherer et al., 2011). The chosen fitness proxies differ in timespan (lifetime vs multiple generations), how they account in their design for the timing of reproduction, and whether they are calculated on a relative versus absolute scale (see Table 1 for a list and description of the proxies). As it has been argued that the best proxy of individual fitness should be one measured over more than one generation (Brommer et al., 2002; Reid et al., 2019), we particularly tested the correlation between lifetime and multi‐generation measures of fitness. Because they all account for some, but not all, difficulties associated with the estimation of fitness, we expected that different proxies would provide different information about individual fitness. Consequently, we also expected that different proxies would be influenced by different factors. Specifically, we expected (a) greater contribution of the demographic context to rate‐sensitive proxies. Because of environmental stochasticity, we expected (b) environmental conditions to affect lifetime proxies more than proxies over multiple generations. Additionally, considering that all individuals living at the same time share similar environmental and demographic conditions, we expected (c) absolute, rather than relative, proxies of fitness to be more affected by such conditions, which would exacerbate the importance of individual conditions. Finally, the environmental and demographic context can be considered as agents of selection, whereas individual traits are the target of selection. By testing both agents and targets of selection, we aimed at comparing the different sources of variation in individual fitness and to see whether different proxies would provide different predictions on selective pressures acting on individual traits.

**TABLE 1:** List of the seven fitness proxies used, their description, consideration of limiting factors, and summary statistics

Fitness proxy	Definition	Time frame	Rate‐sensitive	Relative	Sample size	Average value	Range	Variance
LBS	Lifetime breeding success: Total number of times a female gave birth in her lifetime	•			138	6.56	1–15	0.13
LRS	Lifetime reproductive success: Total number of independent (weaned) offspring produced in an individual's lifetime	•			138	4.47	0–12	8.22
Lambda (λ_i_)	Individual population growth rate: Left eigenvalue associated with the left eigenvector of an age‐classified individual population matrix. Offspring are measured at weaning	•	•		138	1.00	0.00–1.32	0.13
P_life	Contribution to population growth: Individual contribution to population growth rate during its lifetime	•		•	138	0.006	−0.03–0.12	0.001
F2	Contribution to F2: Total number of weaned daughters produced by the daughters of an individual	••			112	1.56	0–15	7.49
Descendance	Next generation descendance: Total number of females descendants alive 2 generations (i.e., 12 years) after the birth of an individual	••	•		106	1.21	0–6	2.24
*r*GC	Relative genetic contributio*n*: Proportional genetic contribution of an individual to the population 2 generations (i.e., 12 years) after the birth of an individual	••	•	•	109	0.012	0.00–0.07	0.0002

*Note*: Dots indicate if each characteristic is true for each proxy. In the “time frame” column, more points indicate a longer time frame (•, lifetime; ••, multiple generations).

## 2 METHODS

### 2.1 Study site, species, and data collection

The study site consists of approximately 38 km^2^ of subalpine meadows and alpine tundra at 1082–2173 m a.s.l on Ram Mountain (52° N, 115° W), Alberta, Canada. From 1974 to 2018, sheep were captured multiple times from late May to late September in a corral trap baited with salt. This population is small and isolated, such that the entire population is monitored, and the sighting probability of females is over 99% (Jorgenson et al., 1997). At first capture, sheep were individually marked with ear tags and/or visual collars and sexed. The age of all individuals was known because they were first captured as lambs or yearlings. At each capture, sheep were weighed with a spring scale to the nearest 250 g. Bighorn sheep mate in late autumn and females give birth to a single offspring the following spring. The lowest recorded age of primiparity at Ram Mountain is 2 years but can range up to 7 years (Martin & Festa‐Bianchet, 2012). After parturition, usually between late May and mid‐June (Renaud et al., 2019), females nurse their lamb until weaning in early autumn prior to the mating season (Festa‐Bianchet, 1988). Female breeding status was determined from field observations of nursing behavior and during captures by udder inspection. Maternities were assigned from direct observations of suckling behavior and confirmed by genetic analyses (Coltman et al., 2005). Weaning success was considered as lamb survival to late September. Since resighting probability is over 99% for females (Jorgenson et al., 1997), ewes were considered dead if unseen at the study site the following year.

### 2.2 Fitness proxies

All analyses were performed in R (R Core Team, 2016). We measured four main fitness proxies: (1) lifetime reproductive success, (2) individual growth rate, (3) individual contribution to population growth and (4) individual contribution to future generations (multi‐generation fitness). Lifetime reproductive success was further divided using the number of lambs born, referred to as lifetime breeding success (LBS) and the number of lambs weaned, referred to as lifetime reproductive success (LRS). LBS and LRS are often measured considering the offspring of both sexes. This is, in part, because the sex of all offspring is often unknown due to the difficulty of, or ethical concerns related to, observing and/or capturing wild vertebrate offspring at a young age. Therefore, here LBS and LRS were calculated considering the offspring of both sexes.

Individual growth rate (λ_i_) corresponds to the dominant eigenvalue associated with the dominant eigenvector of the Leslie population matrix representation on individual life history (Mcgraw & Caswell, 1996). Each individual *i* is considered as a distinct population, with its own age‐specific survival and fertility rates. The individual life cycle graph is constructed using *j* stages. Stage 1 corresponds to the offspring stage (age = 0) and stage *j* corresponds to the last year of observation before death, with the individual population matrix having dimensions *j*x*j*. The subdiagonal of the individual population matrix is then filled with ones, leaving the position *j* at 0. The first row of the matrix represents age‐specific fertilities, which correspond to the number of female offspring produced in each year of life (Mcgraw & Caswell, 1996). See Supplementary Materials S1 for an example. We calculated age‐specific fertility and the resulting λ_i_ using the number of female lambs weaned in a given year. The population growth rate in long‐lived species is mainly concerned with the female component and λ_i_ is thus typically calculated considering only female offspring production (e.g., Brommer et al., 2002; Mcgraw & Caswell, 1996; Zedrosser et al., 2013). Here, we also follow this definition, and weaned lambs of unknown sex (29%) were attributed sex randomly assuming a 1:1 sex ratio at birth as bias in sex ratio has not been found in this species (Blanchard et al., 2005). Calculation of *λ*_*i*_ was performed using the R package *popbio* (Stubben & Milligan, 2007).

Individual contribution to population growth was measured using the method proposed by Coulson et al. (2006). This method, referred to as de‐lifing, calculates the growth of the population (typically considering only females) with and without the survival and reproductive contribution of a focal individual from year *t* to year *t +* 1. The difference represents the individual's relative annual contribution to population growth. The method can also be used to derive an individual's lifetime contribution (P_life) by summing the annual contributions over the lifetime (Alif et al., 2022; Gratten et al., 2008). For this proxy, we only considered female lambs in the reproductive contributions of adult females, here again randomly attributing sex to lambs of unknown sex.

Individual contribution to future generations was calculated using three different proxies. First, we calculated the number of granddaughters produced by each female, i.e., her contribution to the F2 generation (hereafter referred to as “F2”). The number of granddaughters corresponds to the number of weaned female lambs produced by the daughters of a focal female. This fitness proxy could only be calculated for females having produced daughters that died before the end of the study period and that have complete reproductive records. Second, we calculated the number of female descendants still alive two generations later, hereafter referred to as “descendance.” Considering that the generation time in bighorn sheep is about 6 years (Coltman et al., 2003), we calculated the number of female descendants of a focal individual 12 years after her birth. It is important to note that survival senescence begins at age 8, so that few females live longer than 12 years (Loison et al., 1999), and that reproductive senescence begins at age 13 (Martin & Festa‐Bianchet, 2011). Third, following (Bherer et al., 2011), we took advantage of the full pedigree information and the long‐term monitoring program to estimate the relative genetic contribution of females by tracking the alleles of a founder (or here, a focal female) in the descendants (hereafter referred to as the relative genetic contribution, *r*GC) using the pedigree (see Pigeon et al., 2016 for pedigree reconstruction details and description). Briefly, this fitness proxy is measured by summing the transmission probabilities along all the genealogical pathways linking a female and its descendant (Roberts, 1968), i.e., all the potential ways a descendant could have inherited the female's allele. Based on equations presented in Bherer et al. (2011), and considering that an offspring only receives half of the maternal genes, we calculated (Equation 1) the genetic contribution to each descendant, *d* for each focal female, *f* (GC_f,d_), as follows:

(1) $${\mathrm{GC}}_{f,d}=\sum_{i=1}^{p}{\left(\frac{1}{2}\right)}^{g_{i}}$$

where *p* corresponds to the number of genealogical paths linking a focal female to a descendant, *g* is the number of generations separating the focal female *f* and the descendant *d*. Then, using those individual contributions, we derived (Equation 2) the relative genetic contribution of a focal female (*r*GC_f_) to the future population as in Bherer et al. (2011):

(2) $${\mathrm{rGC}}_{f}=\frac{\sum_{d=1}^{n_{r}}{\mathrm{GC}}_{f,d}}{n_{r}}$$

where *n*_*r*_ is the number of individuals in the population two generations (12 years) after the birth of the focal female. We calculated rGC over two generations because the sample size decreased sharply, and most lineages died after two generations (see Supplementary Materials S2 for more details).

For the multi‐generation proxies (F2, descendance, and rGC), we only considered female descendance, i.e., we only tracked daughters and calculated female offspring production. This is because in bighorn sheep males do not provide paternal care and consequently, paternal assignations rely exclusively on genetic analyses. The Ram Mountain bighorn sheep pedigree is deep and well‐connected (Pigeon et al., 2016). It comprises 945 maternal links from 277 dams and 608 paternal links from 128 sires. However, paternal assignations only started in 1988 (Martin et al., 2016). Therefore, considering male and female descendants in the pedigree greatly reduces the number of years we can use for the analyses. We thus decided to focus on female descendants only in the pedigree in the main text. We show in Supplementary Materials S3 the results when considering both male and female descendants in the calculation of the seven fitness proxies (both lifetime and multi‐generation fitness proxies) but acknowledge that those results should be interpreted with caution.

We only estimated the fitness of bighorn sheep females with complete life histories and that have reproduced at least once. These females were monitored from birth to death with known reproductive status at each age. We removed the 16 last cohorts of the study (keeping 26 cohorts of data; 1974–2002) because some females were still alive and we did not have complete reproductive histories (Clutton‐Brock, 1988; Gaillard et al., 2000). For each fitness proxy, description and sample sizes can be found in Table 1, and frequency distributions can be found in Supplementary Materials S4; Figure S6.

### 2.3 Statistical analyses

To evaluate how different fitness proxies correlated with each other, we used Spearman's correlation tests to account for the non‐normal distribution of observations. We conducted pairwise comparisons between fitness proxies with Holm's adjustments using the “psych” R package (Revelle, 2021). While our analyses mainly focused on females that had given birth at least once, we also present pairwise correlations between fitness proxies when considering all females.

For analyses of the determinants of different fitness proxies, determinants included four individual traits, two proxies of environmental conditions, and three proxies of demographic conditions. We considered some determinants over the entire adulthood (≥2 years old) of an individual and at birth since early‐life conditions have long‐lasting effects on fitness in bighorn sheep (Pigeon & Pelletier, 2018). For individual traits, we used age at first reproduction, longevity, body mass at weaning and as a yearling since they are known to affect lifetime reproductive success in large mammals (Festa‐Bianchet et al., 2019; Pigeon et al., 2021; Pigeon & Pelletier, 2018; Zedrosser et al., 2013). Using repeated measurements of sheep mass during the growing season, we adjusted body mass at weaning and yearling mass to September 15 using mixed models (Martin & Pelletier, 2011). As a proxy of prevailing environmental conditions each year, we used the average mass at weaning that year, as it is expected to reflect environmental harshness and availability of resources (Festa‐Bianchet et al., 2004). We used this proxy of environmental conditions at birth and averaged over the adulthood of an individual. For demographic conditions, we used population density (i.e., total female population abundance) at birth and averaged over the adulthood, and population growth rate. We estimated the population growth rate at primiparity because this is when demographic trends may affect individual fitness the most (Brommer et al., 2002). The population growth rate at primiparity was calculated (Equation 3) as follows:

(3) $$\lambda_{t}=\frac{N_{t+1}}{N_{t}},$$

where $\lambda_{t}$ is the annual population growth rate, $N_{t+1}$ is population size the year following primiparity, and $N_{t}$ is population size the year of primiparity. Correlations between determinants varied between 0.04 and 0.78 (Supplementary Materials S4; Figure S7).To evaluate the relative importance of determinants of our fitness proxies, we performed gradient boosting for regressions (GBR) using the h20 R package version 3.32.1.3 (Aiello et al., 2016; LeDell et al., 2021). Gradient boosting, like other machine learning techniques such as random forest analyses, offers many advantages including high predictive performance, low overfitting when adequately tuned, and a strong capacity to model nonlinear relationships and data with non‐normally distributed errors (De'ath, 2007; Elith et al., 2008). Robustness to non‐normality is important given the often highly non‐normal distribution of fitness measures (e.g., lifetime breeding success; Wilson et al., 2005). Also, GBR also offers an efficient and simple assessment of the relative influence of predictor variables on the response variable (Elith et al., 2008).

We fitted a total of seven GBRs, one for each fitness proxy. All included the entire set of previously described predictor variables. GBR hyperparameter tuning was performed with a random discrete grid search as detailed in Supplementary Materials S5. GBRs were validated internally using 5‐fold cross‐validation, which allowed the use of the full extent of available data while avoiding the potential pitfall of “lucky splits” in hold‐out validation (Blum et al., 1999; Yadav & Shukla, 2016). Once hyperparameter tuning was completed, we assessed permutation‐based variable importance using the *DALEXtra* package version 2.1.1 (Maksymiuk et al., 2020) and 20,000 random permutations. Variable permutation importance is the difference in predictive accuracy between a model fit with original data and model fit to data with a randomly permutated predictor variable. For our GBRs, permutation importance was computed as the difference in root mean square error (ΔRMSE) between the original and permutated models (Kazemitabar et al., 2017). Finally, the performance of GBRs was measured using *R*‐squared (LeDell et al., 2021). *R*‐squared reflects the absolute importance of fitness determinants. Therefore, variable importance must be weighed by the *R* ^2^ of a model. For instance, the most important determinant of a model, which only explains 10% of variance is likely less important in absolute terms than the most important determinant of a model, which explains 80% of variance in the response variable.

## 3 RESULTS

### 3.1 Correlations between fitness proxies

**FIGURE 1:**
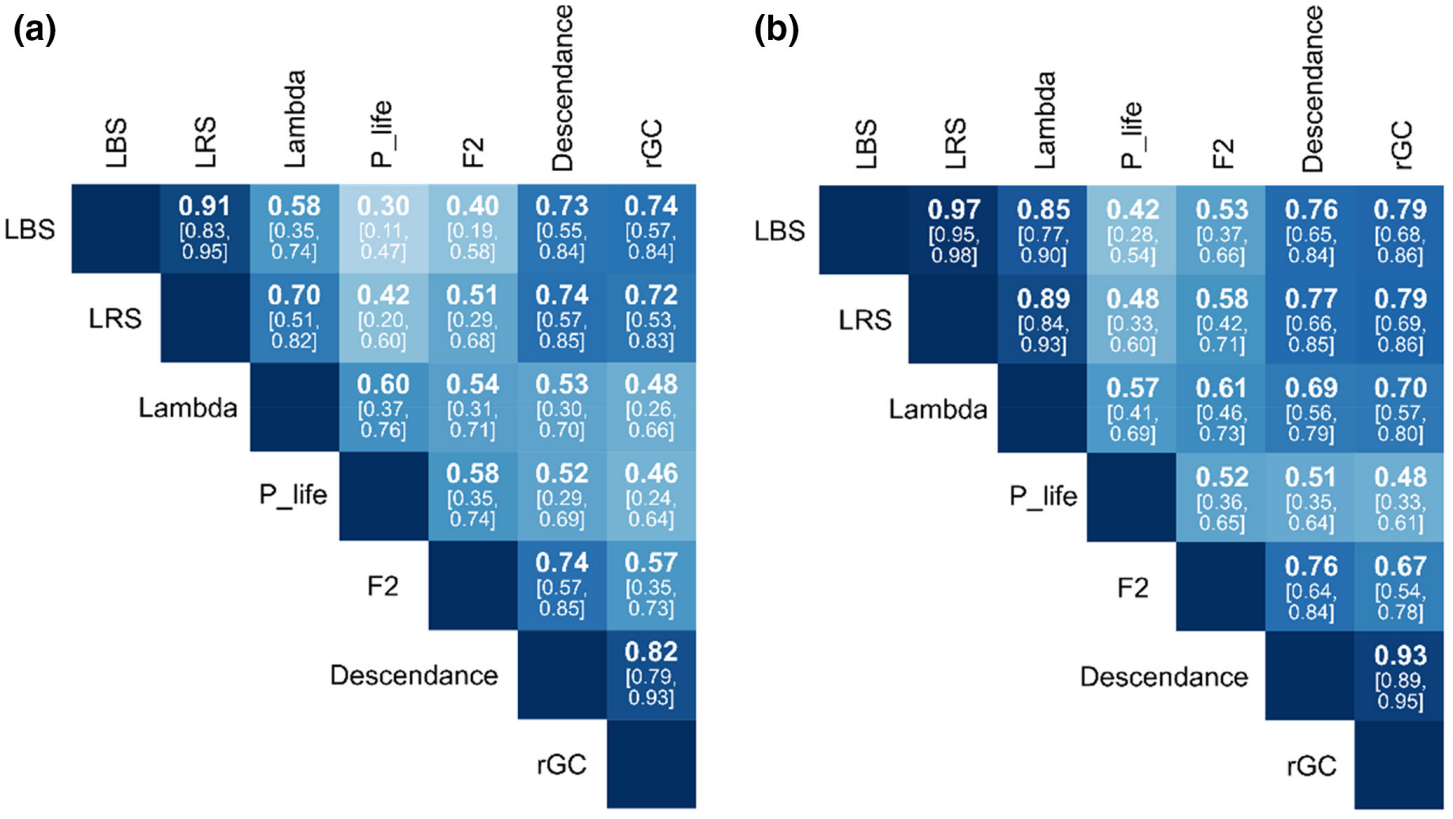
Correlations between the seven proxies of individual fitness estimated on bighorn sheep at Ram Mountain, Canada. Correlations are shown when considering only females that have reproduced once (a) and considering all females in the population (b). Definitions: LBS, lifetime breeding success; LRS, lifetime reproductive success; lambda = individual growth rate (λ), P_life = individual contribution to population growth, F2 = number of granddaughters, descendance = total number of female descendants 2 generations in the future, rGC = relative genetic contribution. Correlation coefficients are shown along with the 95% confidence interval in brackets. Darker colors mean stronger correlations. All correlations are statistically significant at a 0.05 significance level.

All seven proxies could be calculated on 98 females in total. All fitness proxies were positively and significantly correlated with one another (Figure 1a), but most pairwise correlations were only moderate (mean *r* = 0.60, range: 0.30–0.91). Among the lifetime fitness proxies estimated, the strongest correlation was between LBS and LRS. However, λ_i_ and P_life were more strongly correlated with LRS than with LBS. Among the multi‐generation fitness proxies, F2 was more strongly correlated with descendance than with *r*GC. Descendance and *r*GC were highly correlated (*r* = 0.82) with each other. When considering all females—this includes females that have died before starting to reproduce—all pairwise comparisons between fitness proxies increased (Figure 1b). Individual growth rate (λ_i_) became disproportionally correlated with descendance and rGC. Overall, we find a stronger correlation between proxies calculated over similar time frames. Also, LBS and LRS correlated more with the longer‐term proxies descendance and rGC, compared with P_life and lambda.

**FIGURE 2:**
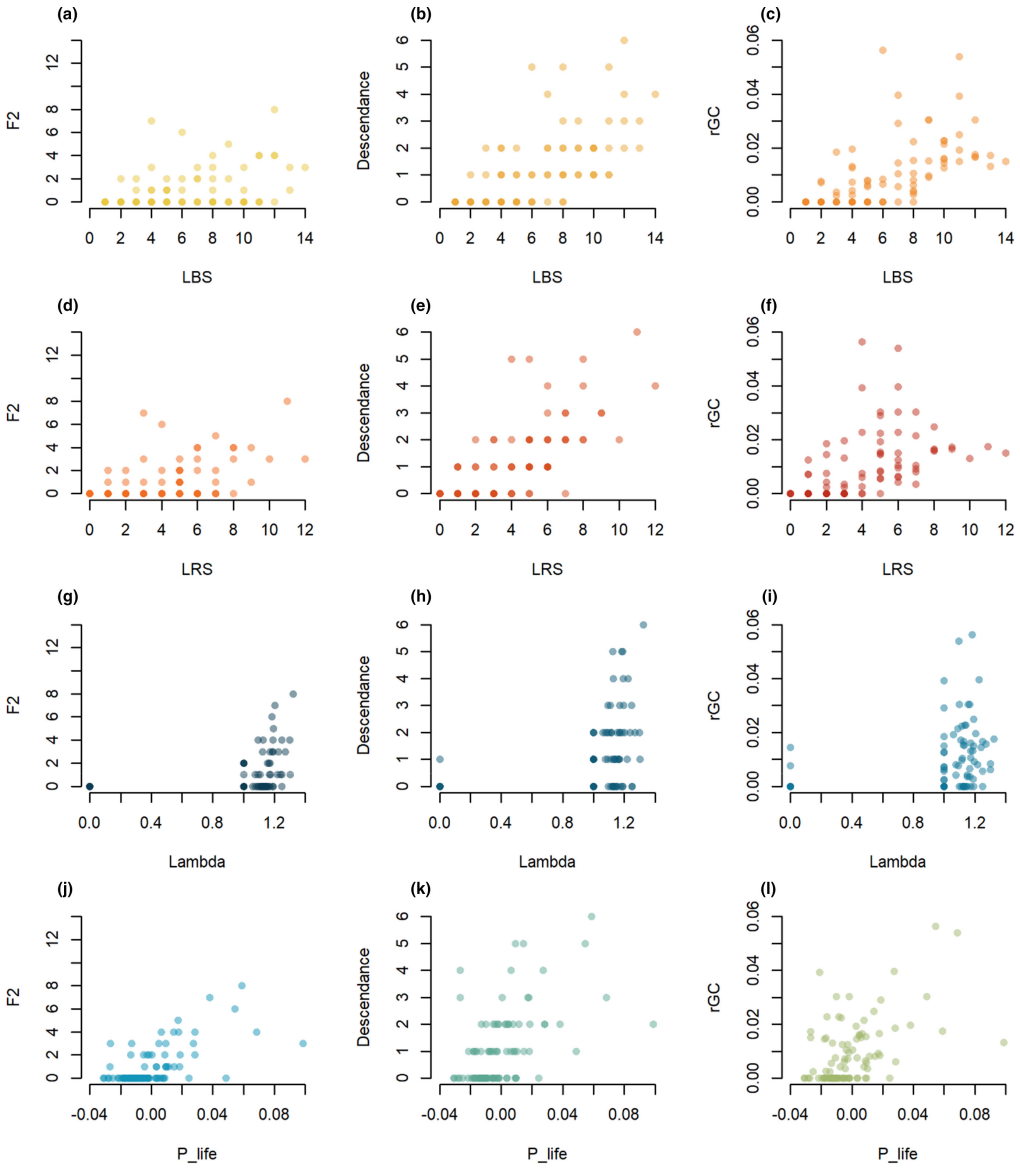
Comparison of the four lifetime (LBS, LRS, lambda, P_life) and the three multi‐generation (F2, descendance, and *r*GC) proxies of fitness in bighorn sheep at Ram Mountain, Canada. See Table 1 for a description of the fitness proxies.

Correlations between lifetime and multi‐generation fitness proxies were strongest between LBS or LRS and descendance (Figure 2b,e) and *r*GC (Figure 2c,f). However, the correlations between LBS or LRS and F2 were weaker (Figure 2a,d). Similarly, lambda (λ_i_) and P_life were moderately correlated with F2 (Figure 2g,j). Overall, P_life showed the weakest correlation with descendance and *r*GC (Figure 2k,l), followed by lambda (λ_i_) (Figure 2h,i). When including male descendance in the calculation of the seven proxies, correlations between proxies were stronger, but a similar pattern was observed overall (Supplementary materials S3).

### 3.2 Determinants of fitness

**FIGURE 3:**
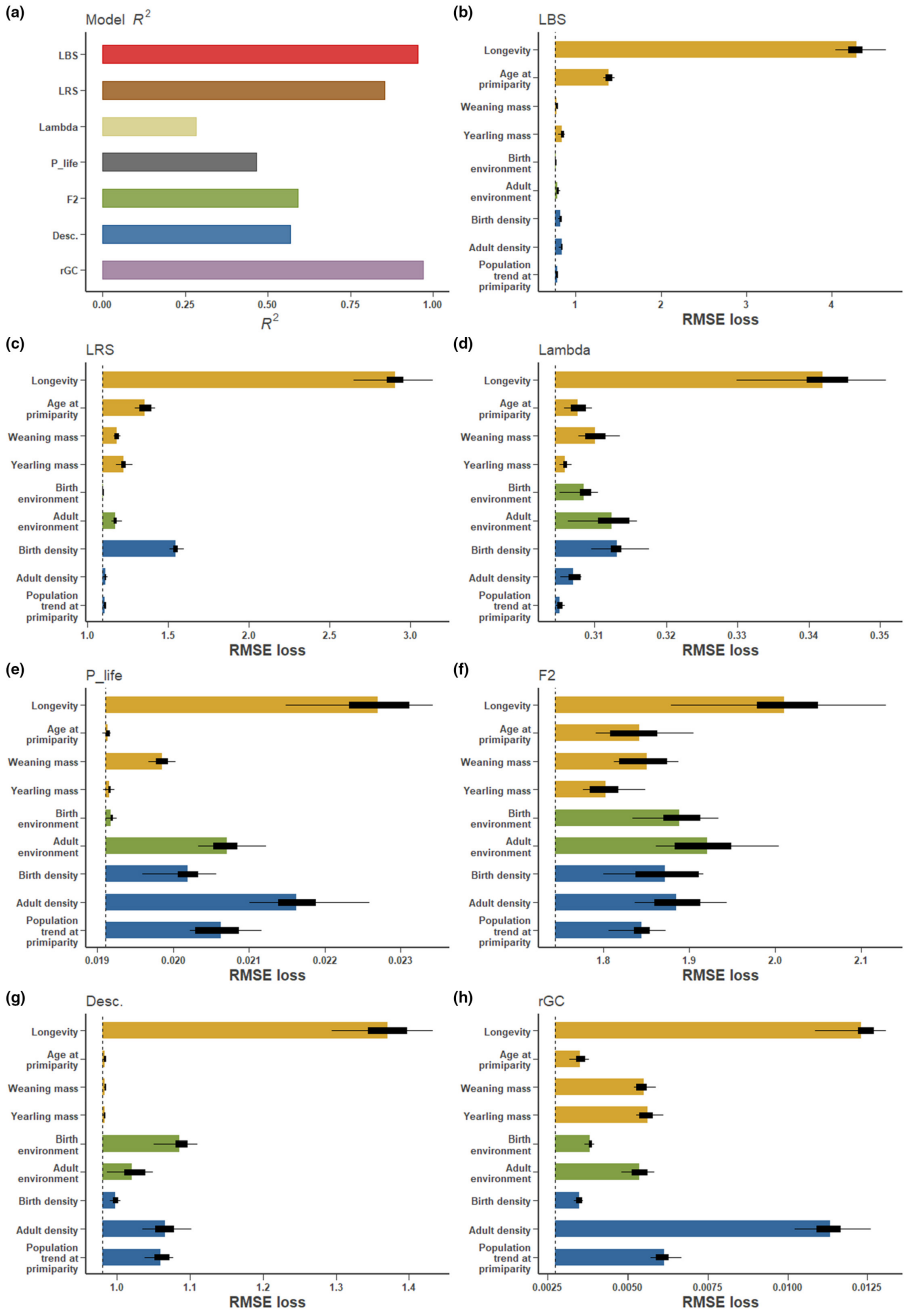
Model *R*‐squared of gradient boosted regressions fitted to assess the determinants of different fitness proxies of bighorn sheep at Ram Mountain, Canada, are shown in panel (a) permutation‐based importance of the determinants of fitness proxies are shown in panels (b–h) Golden bars represent individual life‐history determinants, green bars represent environmental determinants and blue bars represent demographic. Determinants. In panels b–h, the vertically dotted line represents the root mean square error (RMSE) from the model fitted to the original data whereas bars extend to the mean RMSE of models fitted after permutating the predictor variable 20,000 times. Bar length thus represents the increase in RMSE after permutation. Boxplots represent the distribution of RMSE values of models fitted after permutations.

The GBRs explained a moderate to high proportion of variance in our fitness proxies as judged by R‐squared values (range: 0.28–0.96; Figure 3a). The proportion of variance explained was above 0.75 only for LBS, LRS and *r*GC. The variance explained was particularly low for λ_i_ and P_life.

Age at death was a relatively important individual life‐history determinant for all fitness proxies (Figure 3b–h). For LBS and LRS, age at death was arguably the sole critical fitness determinant (Figure 3b,c). This was especially true for LBS where other determinants were negligible relative to age at death. The other individual traits, which were age at primiparity, weaning mass, and yearling mass, were generally unimportant relative to the most important determinants (Figure 3), except for F2 and rGC. Another notable exception with slightly higher relative importance was weaning mass for P_life (Figure 3e). Our proxies of environmental conditions at birth and over adult life were important determinants for λ_i_, P_life, F2, descendance, and rGC (Figure 3d–h). Adult environment was more important than birth environment in most cases except for descendance. Demographic conditions were relatively important determinants of LRS, lambda, F2, and descendance but were especially important relative to other determinants for P_life and rGC (Figure 3d–g). For both P_life and rGC, adult density was the second most important determinant nearing the importance of age at death. Among the individual proxies, the relative importance of age at primiparity was greater for LBS and LRS, but weaning mass was relatively more important for lambda, P_life, F2, and rGC. This suggests that depending on the fitness proxy used, we would predict higher selective pressure on different individual traits. General tendencies of results on the determinants of fitness proxies remained largely unchanged when male descendants were considered in the calculation of λ_i_, P_life, F2, descendance, and rGC (Supplementary Material S3; Figure S5).

## 4 DISCUSSION

Our main objective was to compare proxies of individual fitness and investigate the determinants of individual fitness in female bighorn sheep. By considering seven fitness proxies that fundamentally differ in their consideration of the timing of reproduction, environmental conditions, and demographic context, our analyses showed that fitness proxies cannot be used interchangeably. Although all empirical estimates of fitness proxies were significantly correlated with one another, their associations were not always strong, and most importantly, their determinants differed substantially. In most cases, the dominant determinant of individual fitness in bighorn sheep was longevity but the relative importance of other determinants, such as environmental conditions and demographic context varied between the different proxies used. Our results on bighorn sheep thus suggest that different proxies could lead to a different interpretation of selective pressures at play and potentially divergent predictions of evolutionary outcomes.

Pairwise correlations of the seven fitness proxies were all positive and significant, but the magnitudes of the correlations varied considerably. LBS, LRS, and λ_i_ were strongly correlated, though correlations were weaker than most reported in previous studies on vertebrates (Brommer et al., 2004; Käär & Jokela, 1998; MacColl & Hatchwell, 2004; Mcgraw & Caswell, 1996). The relationship between λ_i_ and LRS was curvilinear, as previously found by Brommer et al. (2002) and Lardy et al. (2015). P_life was only very weakly correlated with LBS and LRS, potentially because of a lesser dependence on longevity and greater integration of population dynamics. This result contrasts with findings on Soay sheep, *Ovis aries* (Coulson et al., 2006) but may be explained by larger fluctuations in the Ram Mountain bighorn sheep population over the study period (Supplementary Materials S4; Figure S8).

Corroborating previous studies (Brommer et al., 2004; Reid et al., 2019), we found that among the lifetime fitness proxies, lifetime reproductive success was most correlated with genetic contributions over multiple generations. In house sparrows, however, de‐lifing and individual growth rate were similarly correlated with individual long‐term reproductive value but only when considering offspring at the recruits stage (Alif et al., 2022). Nevertheless, our results show that lifetime fitness proxies are relatively poor indicators of genetic contributions to more than one generation in the future, which further corroborates Brommer et al. (2004), and Reid et al. (2019). This finding supports the idea outlined by Reid et al. (2019), that the further we look into the future, the correlations between lifetime fitness and representation of genetic contributions becomes weaker. Our results thus underline the need to find consensus on the best way of approximating long‐term genetic contributions from single‐generation data.

Unsurprisingly given imperfect and moderate correlations among fitness proxies, the relative importance of determinants for explaining variation in bighorn sheep fitness differed considerably from one proxy to another. A notable exception was for longevity, which was the most important determinant of all seven fitness proxies considered. For LBS and LRS, longevity was arguably the only determinant of individual variation in fitness. The importance of longevity was inevitable in bighorn sheep since ewes can only produce one lamb per year and rarely skip a reproductive event. Indeed, females ≥3 years lactate >90% of years (Festa‐Bianchet et al., 2019; Pigeon & Pelletier, 2018), leaving little room for inter‐individual variation in annual reproduction (Festa‐Bianchet et al., 2019). Therefore, most variation in fitness in bighorn sheep is driven by the number of reproduction opportunities individuals experience, hence longevity. Such a pattern is expected for all species, like bighorn sheep, that are positioned on the slow end of the slow‐fast continuum of life histories (Gaillard et al., 2016). For species with a slow life history, longevity could thus arguably be used as a stand‐alone proxy of fitness when tracking reproduction is not possible. A comparison with species with higher reproductive rates, i.e., species on the fast end of the continuum, would most certainly be instructive.

Relative proxies and those accounting for the timing of reproduction and the demographic context revealed other determinants of variation in individual fitness. Unexpectedly, age at primiparity was not important in explaining variation in λ_i_ (Mcgraw & Caswell, 1996; Rubach et al., 2020). Age at primiparity, however, was very important in explaining λ_i_ when male offspring where also considered (Supplementary Materials S3; Figure S5d). Weaning mass, adult environmental conditions, and birth density were relatively strong determinants of λ_i_. In a study on brown bears, yearling mass was also identified as the second most important determinant of λ_i_ after longevity (Zedrosser et al., 2013). As expected, variation in P_life was largely driven by population density, both at birth and during the adulthood. The pathways linking population density to the life histories of bighorn sheep at Ram Mountain are complex (Pigeon & Pelletier, 2018), and fitness proxies other than LBS and LRS may better capture these complex dynamics. The sensitivity of P_life to density was expected as by design P_life is constructed relative to population abundance and the same is true for rGC. However, P_life and rGC were also both sensitive to population growth rate, suggesting that how well an individual performs relative to others depends on whether the population is growing or declining. Contrary to our expectation, we found that individual characteristics contributed less to variation in individual fitness over multiple generations, but environmental conditions and demographic context remained important. Our results suggest that fitness over multiple generations is driven by a combination of the most important variables predicting different lifetime fitness proxies.

To understand and predict evolutionary dynamics, we ought to rely on some measure of fitness. Indeed, selection gradients typically rely on the regression of one phenotypic trait on a single proxy of individual fitness (Reid et al., 2019). But when choosing a fitness proxy, we are faced with a dilemma: there is no agreement as to which fitness proxy is the best. In many situations, field limitations constrain the choice of fitness proxy that can be used by researchers. However, provided having sufficient data to do so, many advocate that fitness is best reflected by the number of descendants more than one generation in the future (e.g., Brommer et al., 2002; Reid et al., 2019). Measuring fitness over multiple generations nevertheless raises some concerns. The more we look into the future, the more parental and offspring fitness become intertwined (Wilson et al., 2005). This can become problematic when estimating selection, as the response to selection becomes a component of fitness at longer timescales. Instead, some argue that as selection operates on short time frames, evolutionary predictions should be based on short‐term (e.g., annual) fitness (Alif et al., 2022; Coulson et al., 2006). Evolutionary ecologists have also been urged to lean towards a more demographic consideration of fitness, and several additional demography‐sensitive methods of measuring annual and lifetime fitness are available (Metcalf & Pavard, 2007).

Here we focussed on females that have reproduced at least once in their lifetime, as in, e.g., Brommer et al. (2002) and Viblanc et al. (2022), since long‐term monitoring programs typically target mature females. In Figure 1b, we showed correlations between fitness proxies measured on the entire female population, which includes females that have died before reaching reproductive age and females that reached reproductive age but never reproduced. The distribution of fitness including all females is highly zero‐inflated (Supplementary Materials S6; Figure S9) and, as a result, correlations between all seven proxies are strengthened. However, the main conclusions remain. We could not perform the second analysis on the determinants of fitness using all females as most of the determinants were not measured at birth. For example, age at first reproduction requires that the individual reach reproductive age. Nevertheless, we can speculate that having added those females, longevity would have become relatively even more important for all proxies, especially since whether an individual survives to reproductive age is often only a question of luck (Snyder et al., 2021).

Our approach, based on multiple fitness proxies with contrasting definitions, showed that different proxies of fitness do not carry the same information. Depending on the fitness proxy used, selection and predictions of evolutionary change may thus vary. For instance, here we would predict greater selective pressure on age at primiparity using LBS and LRS but greater selective pressure on weaning mass using λ_i_, P_life, F2, and rGC. This suggests that fitness proxies should not be used interchangeably and makes comparisons between studies estimating fitness differently impractical. It also raises the critical question: which proxy should be used? While our study does not provide an answer to this, it is illuminating in that it suggests that different proxies may be used in different contexts. For instance, for a population with the unstable population growth rate, proxies sensitive to demography might be preferable. In bighorn sheep, lifetime fitness proxies provide an incomplete picture of the long‐term story. Therefore, unless we have a clear a priori understanding of the specific context of our study system (e.g., the timing of reproduction is important in nonequilibrium populations; Brommer et al., 2002; Stearns, 1992), the subtleties of different fitness measures call for the consideration of contrasting fitness proxies, when possible, as each proxy may provide a different piece of the puzzle.

## AUTHOR CONTRIBUTIONS

**Joanie Van de Walle:** Conceptualization (lead); data curation (lead); formal analysis (lead); methodology (lead); validation (lead); writing – original draft (lead); writing – review and editing (lead). **Benjamin Larue:** Conceptualization (lead); data curation (lead); formal analysis (lead); methodology (lead); validation (lead); writing – original draft (lead); writing – review and editing (lead). **Gabriel Pigeon:** Formal analysis (supporting); methodology (supporting); writing – original draft (supporting); writing – review and editing (supporting). **Fanie Pelletier:** Conceptualization (lead); funding acquisition (lead); project administration (lead); resources (lead); supervision (lead); writing – original draft (supporting); writing – review and editing (supporting).

## CONFLICT OF INTEREST

The authors declare no competing interests.

## Supporting information


**Appendix S1:** Supporting Information

## Data Availability

The data used to perform the statistical analyses and produce the figures in the manuscript will be uploaded to a public repository (e.g., Dryad) upon acceptance of the manuscript.
