# Supplementary material for: Different proxies, different stories? Imperfect correlations and different determinants of fitness in bighorn sheep
Source: Ecol Evol. 2022 Dec 8;12(12):e9582. doi: 10.1002/ece3.9582 (PMC9731912; doi:10.1002/ece3.9582)
Supplement: Supplementary file 1 — Appendix S1: Supporting Information [file ECE3-12-e9582-s001.docx]

**Title:** Different proxies, different stories? Imperfect correlations and different determinants of fitness in bighorn sheep

**Authors**: Joanie Van de Walle^1^*, Benjamin Larue^2^*, Gabriel Pigeon^3^, and Fanie Pelletier^2^

^1^Biology Department, Woods Hole Oceanographic Institution, Woods Hole, MA, USA

^2^Département de Biologie, Université de Sherbrooke, Sherbrooke, Québec, Canada

^3^Institut de recherche sur les forêts, Université du Québec en Abitibi-Témiscamingue, Rouyn-Noranda, Québec, Canada

**Supplementary materials**

**Supplementary materials S1:** Example of calculation of individual growth rate:


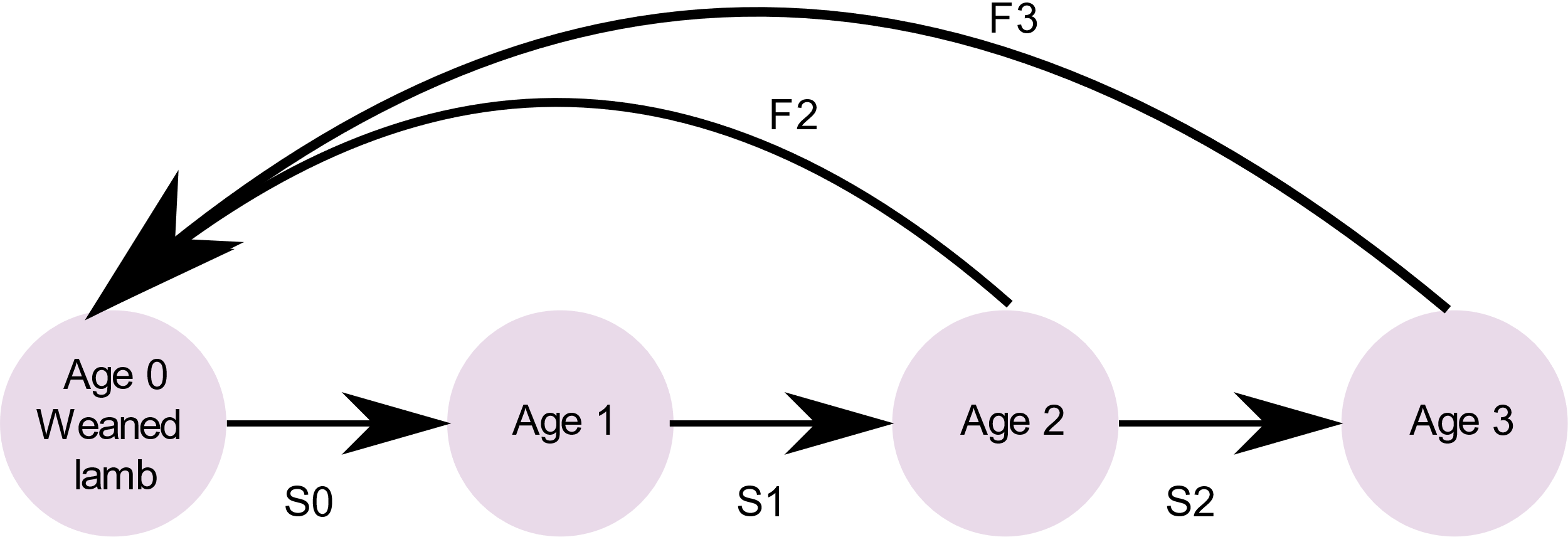


**Figure S1.** The life cycle of an individual that died at the age of four years old (i.e., survived until the age of three years-old). In this example, which is based on post-breeding census, the individual mates at the age of two and three years-old and give birth to a lamb at the age of three and four years-old.

The individual life cycle graph presented in Fig. S1 can be translated into a projection matrix **A***_i_*, from which, alike in the case of an actual population, the dominant eigenvalue (λ_i_) can be extracted analytically (Mcgraw and Caswell 1996; Caswell 2001).

$$A_{i}=\left( \begin{matrix} 0 & 0 & F_{2} & F_{3} \\ S_{0} & 0 & 0 & 0 \\ 0 & S_{1} & 0 & 0 \\ 0 & 0 & S_{2} & 0 \end{matrix} \right)$$

**Supplementary materials S2:** rGC calculation over more than two generations

In the main text, rGC was calculated by tracking the alleles of a founder in the descendance over time. In the main text, we chose to calculate rGC after two generations, but we could have calculated rGC over more generations. Considering that our study spans 54 years and that bighorn sheep generation time is 6 years, the maximum number of generations over which we can calculate rGC is 9. As we increase the number of generations, we also drastically reduce sample size. For instance, using only female lineages, sample size drops to 57 females after 5 generations (Table S1). In addition, after 5 generations rGC becomes 0 for 95% of the females considered here (Fig. S2). For the 57 females having information on 5 generations, the correlation between rGC calculated at 2, 3, 4 and 5 generations is also weak (2 vs 3 generations: r^2^ = 0.5, 3 vs 4 generations: r^2^ = 0.40, 4 vs 5 generations: r^2^=0.39). When we include male lineages in female descendance, we have to remove cohorts before 1988 as DNA assignment of fathers only started at that year. This resulted in only 30 years of data, and we could only track 5 generations. Sample size drops to 0 females after 5 generations and 32 females after 4 generations. The proportion of females having rGC=0 after 4 generations is 0.78. Based on the 32 females with information up to 4 generations, the correlation between rGC calculated at 2, 3, and 4 generations becomes weaker as the number of generations increases (2 vs 3 generations: r^2^ = 0.71, 3 vs 4 generations: r^2^ = 0.65). Therefore, we are unfortunately limited in our ability to measure rGC over more than 2 generations. The very large proportion of females having rGC values of 0 after some generations suggests that most lineages tend to disappear, a phenomenon that is probably frequently observed in natural populations with fluctuating population densities, such as the Ram mountain bighorn sheep population.

**Table S1.** Mean values of rGC, the proportion of females having a rGC = 0 and sample size for rGC calculated over 2, 3, 4 and 5 generations using female-only, and male and female lineages in the descendance, respectively. For a sample of females for which rGC could be calculated over 5 generations using female lineages only, the correlation between rGC calculated over 2 generations and over 3, 4, and 5 generations using female-only lineages are also presented. As sample size over 5 generations is zero using male and female lineages, similar correlations are presented for a sample of females for which rGC could be calculated over 4 generations.

|  | Proportion rGC = 0 | Sample size | Correlation with rGC calculated over 2 generations |
| --- | --- | --- | --- |
| **Female lineages only** |  |  | **based on 57 females with rGC calculated over 5 genations* |
| Two generations | 0.39 | 109 | NA |
| Three generations | 0.7 | 104 | 0.5 |
| Four generations | 0.91 | 89 | 0.4 |
| Five generations | 0.95 | 57 | 0.39 |
|  |  |  |  |
| **Male and female lineages** |  |  | **based on 32 females with rGC calculated over 4 generations* |
| Twogenerations | 0.5 | 52 | NA |
| Three generations | 0.66 | 47 | 0.71 |
| Four generations | 0.78 | 32 | 0.65 |
| Five generations | NA | 0 |  |


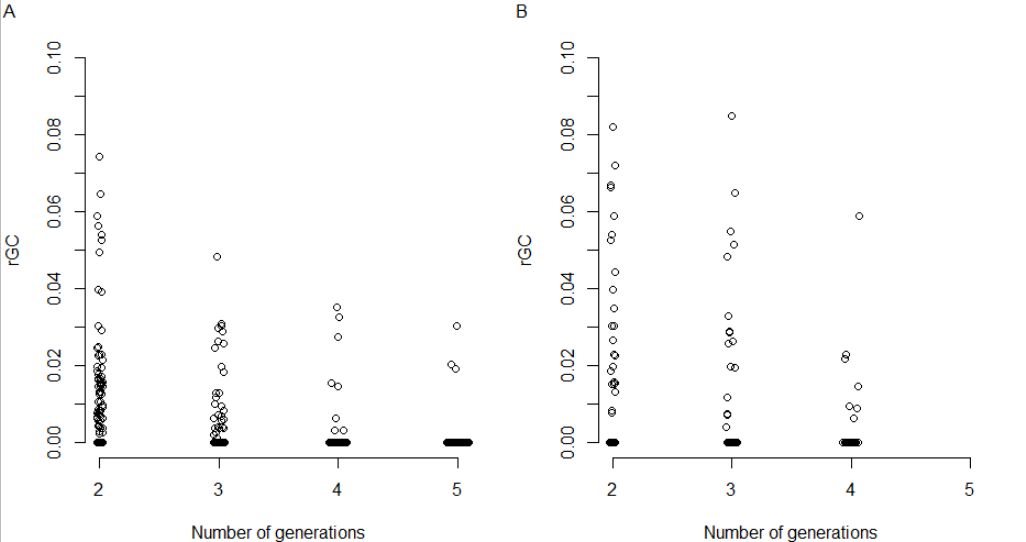


**Figure S2. rGC calculated at 2, 3, 4 and 5 generations for female lineages (A) and female and male lineages (B).**

**Supplementary materials S3:** Impact of considering offspring of both sexes in the calculation of the fitness proxies

In the main text, we focused on female descendance only because some paternities are unknown in the pedigree. Indeed, genetic sampling to assess paternities only started in 1988, whereas maternities were known for the entire study period. This greatly reduces sample size (see main text for details). However, here we tested the strength of the correlation between the seven proxies considered in the main analyses, but this time including male descendance in the calculation of each proxy, acknowledging that those results should be interpreted with caution. The two proxies lifetime breeding success (LBS) and lifetime reproductive success (LRS) already included male and female offspring. Lambda is typically calculated using fertilities in terms of female offspring production, but here we also considered the possibility of including male offspring in maternal fertilities. Similarly, P_life was recalculated considering male production in annual contributions. For the recalculation of the longer-term fitness proxies (F2, Descendance and rGC), we followed for each focal female its lineages through male and female offspring. F2 includes both male and female grand offspring, and Descendance and rGC include all male and female descendants.

The correlation between fitness proxies ranged from 0.59 to 0.91 (mean = 0.73). All correlations are statistically significant at a 0.05 significance level. The correlation between most fitness proxies is strengthened by the inclusion of male descendants in the analyses (Fig. S2-S3), except for the correlation between lambda and P_life which remained unchanged. Overall, we find stronger correlations between proxies calculated over similar timeframes. Also, LBS and LRS correlated more with the longer-term proxies Descendance and rGC, compared to P_life and lambda (Fig. S2-S3).

The gradient boosted regressions explained a high proportion of variance in our fitness proxies that included males as judged by R-squared values (range: 0.63 - 0.96; Fig. S4A). The proportion of variance explained was higher when males were included in the calculation of the fitness proxy for λ_i_, P_life, F2 and Descendance. This increase in R-squared values is likely because of increased randomness when removing males from the calculation of fitness proxies.

Age at death was still an important individual life-history determinant for all fitness proxies (Fig. S4B-H). The other individual life-history determinants, which were age at primiparity, weaning mass and yearling mass, were more important when males were included in the calculation of λ_i_, P_life and Descendance. A notable change from Fig. 3 is the high increase in the importance of age at primiparity for λ_i_. This result is closer to expectations as λ_i_ is thought to give more importance to the timing of reproduction (Mcgraw and Caswell 1996). The importance of demographic conditions also increased for λ_i_, Descendance, P_life and rGC when males were included. For P_life, adult density now nears the importance of age at death whereas for rGC, adult density is now slightly more important than age at death (Fig. S4G-H).


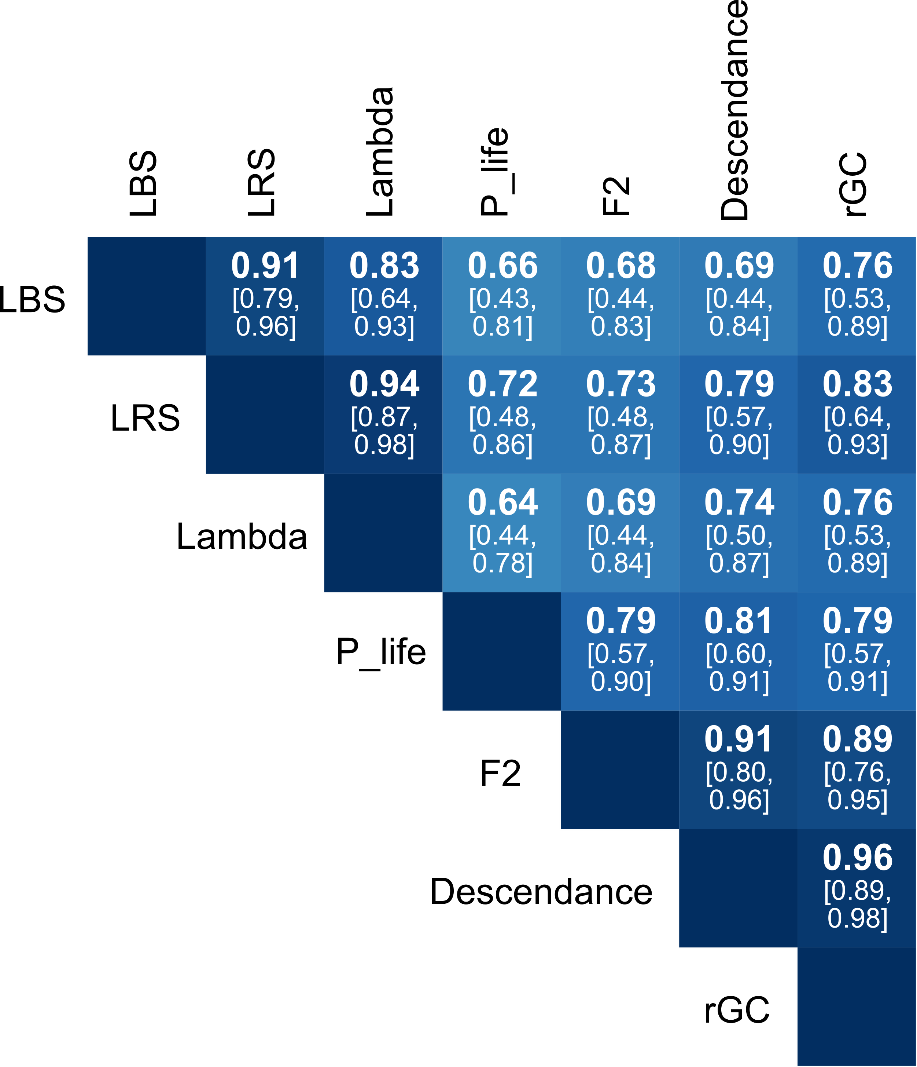


**Figure S3.** Correlations between the seven proxies of individual fitness estimated including also male descendance on bighorn sheep at Ram Mountain, Canada. Definitions: LBS = Lifetime Breeding Success, LRS = Lifetime Reproductive Success, Lambda = individual growth rate (λ), P_life = individual contribution to population growth, F2 = number of granddaughters, Descendance = total number of female descendants 2 generations in the future, rGC = relative genetic contribution. Correlation coefficients are shown along with the 95% confidence interval in brackets. Darker colors mean stronger correlations.

**
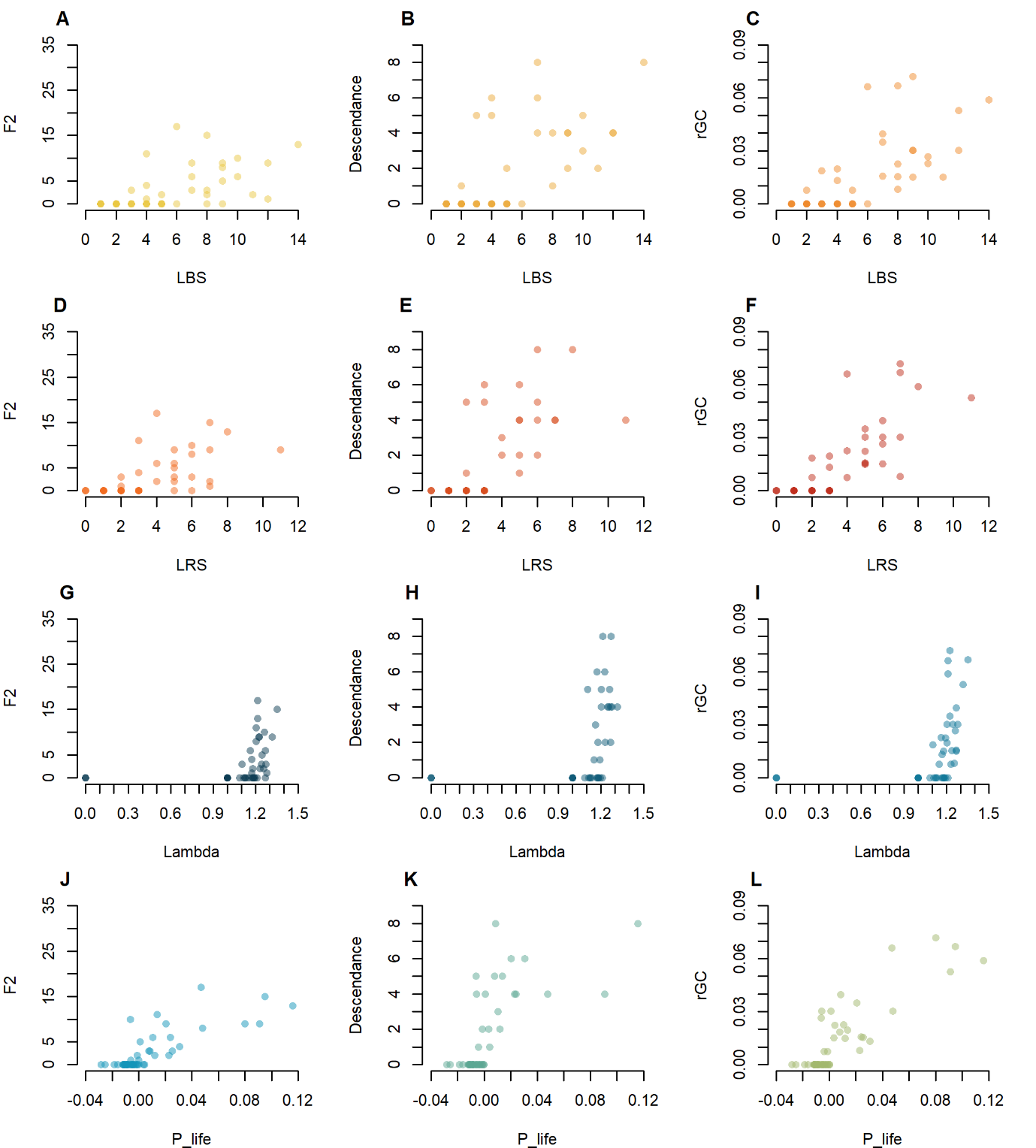
**

**Figure S4.** Comparison of the four lifetime (LBS, LRS, lambda, P_life) and the three multigeneration (F2, Descendance and *r*GC) proxies of fitness in bighorn sheep at Ram Mountain, Canada, including also male descendance. See Table 1 for a description of the fitness proxies.


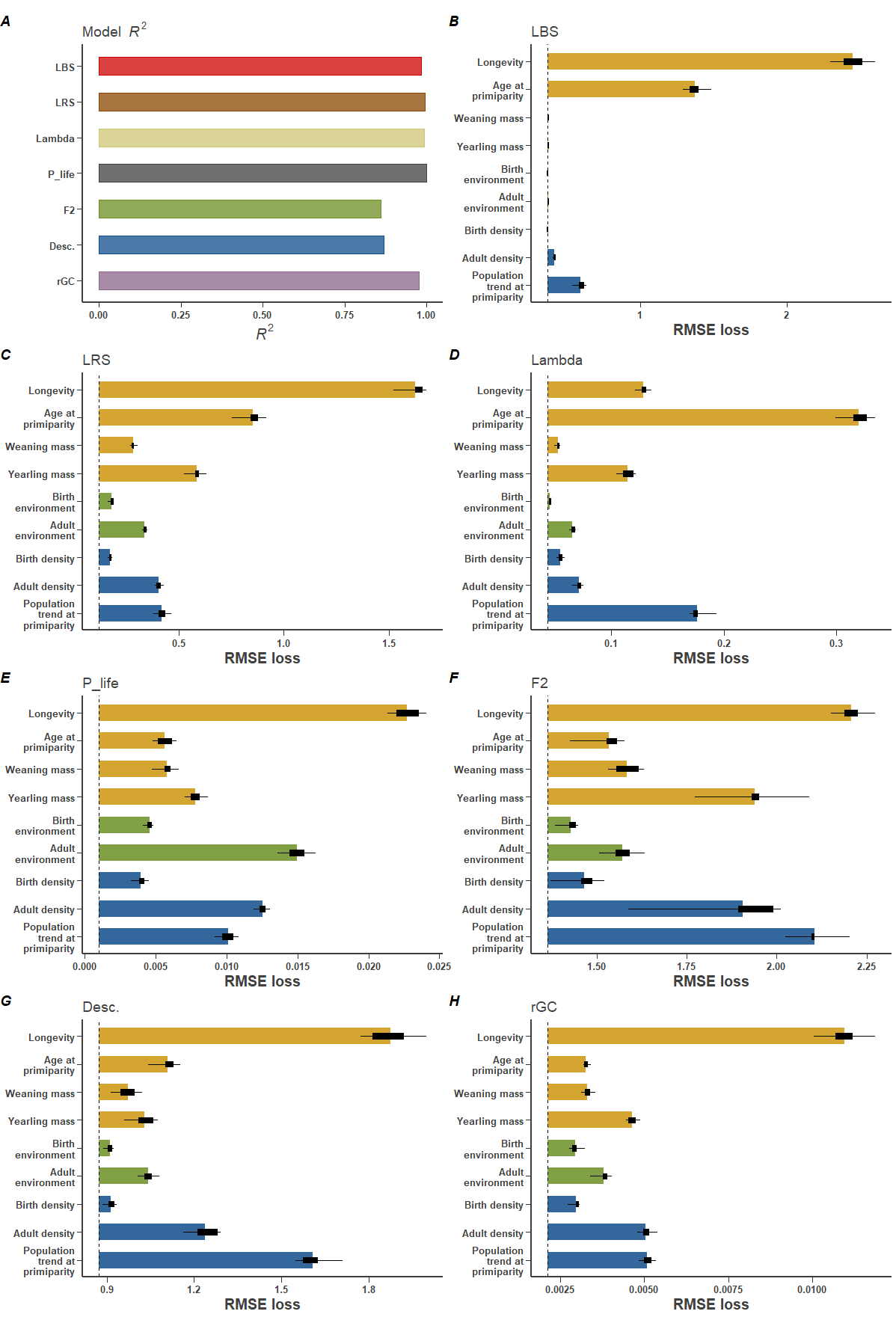


**Figure S5.** Visual representation of the determinants of fitness proxies calculated using both female and male descendance of bighorn sheep at Ram Mountain, Canada. Model R-squared of gradient boosted regressions are shown in panel A. Permutation-based importance of the determinants of fitness proxies are shown in panels B-H. Golden bars represent individual life-history determinants, green bars represent environmental determinants and blue bars represent demographic. determinants. In panels B- H, the vertical dotted line represents the root mean square error (RMSE) from the model fitted to the original data whereas bars extend to the mean RMSE of models fitted after permutating the predictor variable 20 000 times. Bar length thus represents increase in RMSE after permutation. Boxplots represent the distribution of RMSE values of models fitted after permutations.

**Supplementary Materials S4:** Supplementary Figures

**
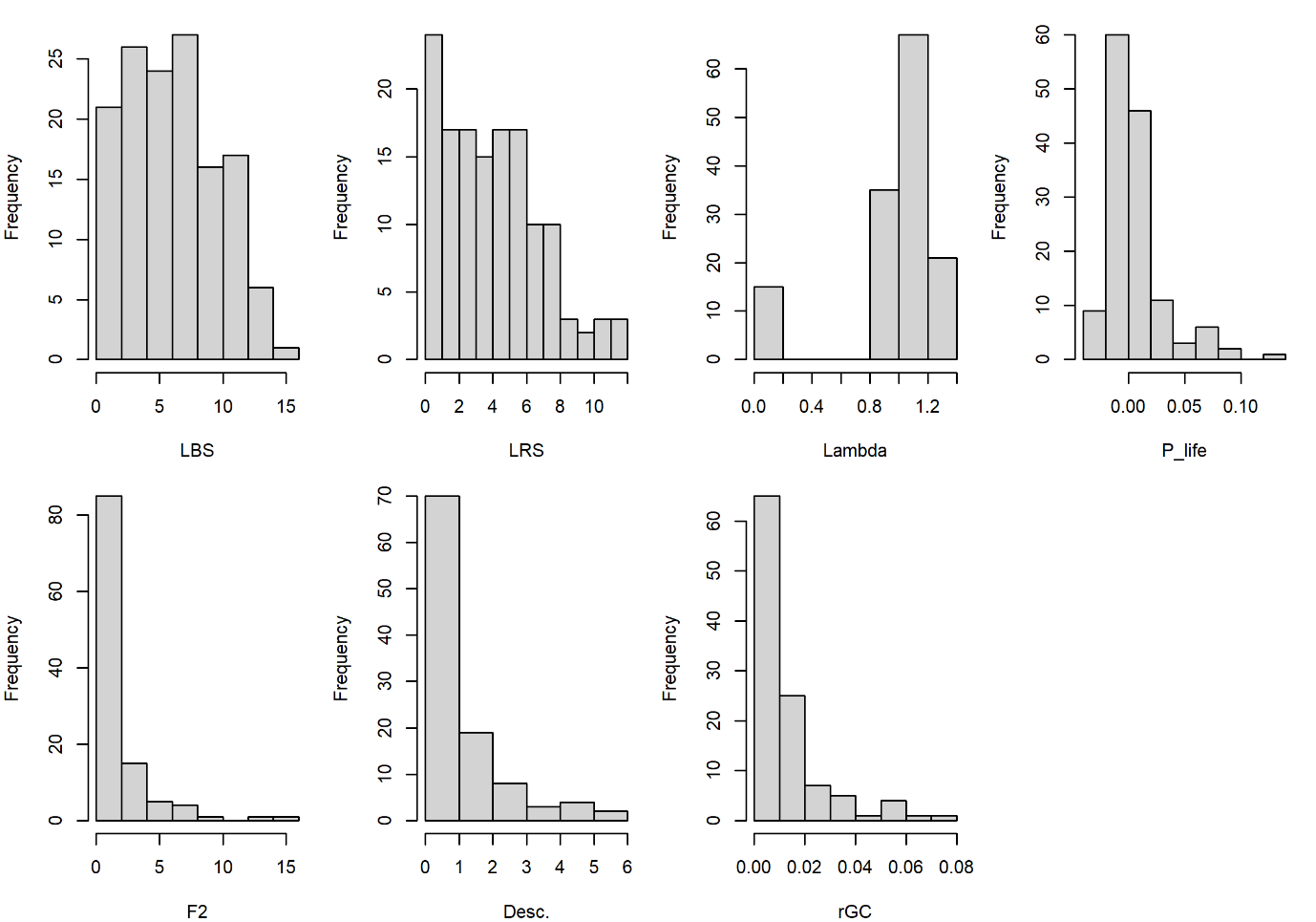
**

**Figure S6:** Histograms showing the distribution of the seven different proxies calculated on female bighorn sheep from Ram Mountain, Canada.


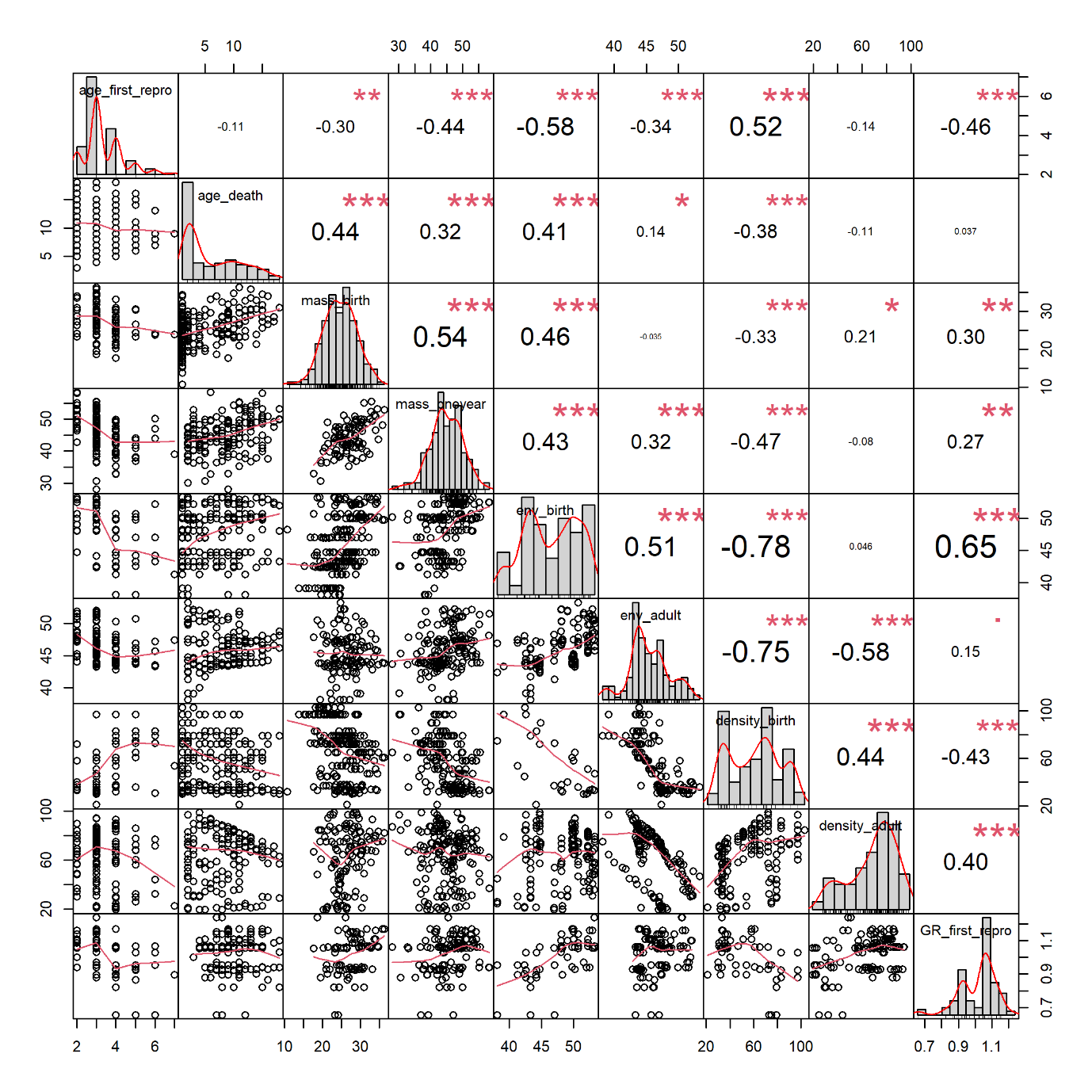


**Figure S7.** Correlation between the predictor variables used in the Gradient Boosting Regressions to determine the relative importance of fitness determinants. Definitions: “age_first_reproduction” = Age at primiparity, “age_death” = Longevity, “mass_birth” = Weaning mass, i.e. mass the year or birth taken at weaning (kg), “mass_oneyear” = Yearling mass, i.e. mass taken at one year of age, “env_birth” = Birth environment, “env_adult” = Adult environment, “density_birth” = Birth density, “density_adult” = Adult density, “GR_first_repro” = Population trend at primiparity.


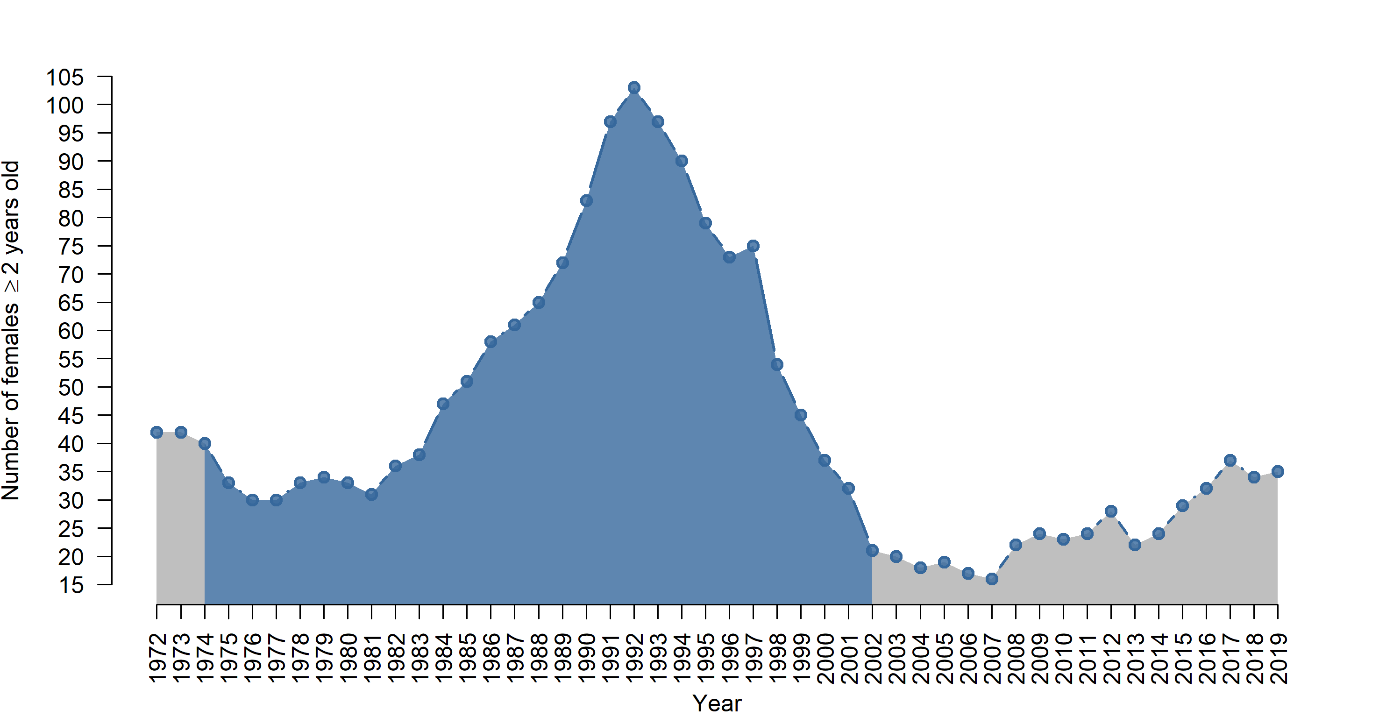


**Figure S8**: Number of bighorn ewes ≥2 years old at Ram Mountain during the period 1972-2019. In blue are cohorts considered in the analyses. The last 16 cohorts were not considered to avoid bias in individual fitness estimations (Gaillard et al. 2000) because some individuals were still alive in 2019.

**Supplementary material S5:** Hyper-parameter tuning for gradient boosted regressions of fitness proxies.

We used a random discrete grid search to determine the combination of multiple hyper-parameters that optimizes cross-validated predictions in our gradient boosted regressions of fitness proxies (Bergstra and Bengio 2012). R code for hyper-parameter tuning and fitting of gradient boosting regressions is shared as supplementary materials. In random grid search, models are fit using random combinations of parameters within the specified grid. This option was chosen because we searched over a very large grid (>1000 different hyperparameter combinations) which would have been extremely computationally demanding in a cartesian grid search approach (i.e., a model is fit to every combination of values within the grid). Random grid search was done using the h2o.grid function from the *h20* R package version 3.32.1.3 (Aiello et al. 2016; LeDell et al. 2021). View Peck (2016) for additional information on hyperparameter optimization with the h2o package. For models fit during the random grid search, we set the number of trees to 10 000 but used early stopping to avoid overfitting. Early stopping occurred when 5-fold cross-validation RMSE improvement after ten training rounds was <1/100 of the range of the response variable. This approach ensured that the stopping tolerance was scaled to all the response variables which greatly differed in range (Table 1). The hyper-parameter grid on which the random discrete tuning was done was created using the seq function from base R. There are three main arguments to the seq function: 1. the starting value of a sequence, 2. the end value of a sequence, and 3. the increment by which numbers are generated between the two ends of the sequence. For instance, seq(0, 1, 0.1) is the sequence of numbers between zero and one by increments of 0.1 which also includes zero and one. The sequence for the seven hyper-parameters included in our final hyper-parameter grid was the following: maximum tree depth = seq(1, 20, 1), minimum number of observations for a leaf in order to split = seq(2, 30, 1), learning rate = seq(0.001, 0.5, 0.001), row sampling rate = seq(0.3, 1, 0.05), column sampling rate = seq(0.3, 1, 0.05), column sampling rate for each tree = seq(0.3, 1, 0.05) and the number of bins to be included for categorical predictors = seq(100, 1000, 100).

**Supplementary material S6:** Impact of considering only females that have reproduced once.

Here, we tested the strength of the correlation between the seven proxies considered in the analyses, but this time including females that have never breed (lifetime breeding success, LBS = 0). Including all females resulted in a highly zero-inflated distribution for all proxies (Fig. S8). The correlation between fitness proxies ranged from 0.42 to 0.97 (mean = 0.68). All correlations are statistically significant at a 0.05 significance level. The correlation between most fitness proxies is strengthened by the inclusion of all females in the analyses (Fig. S9).


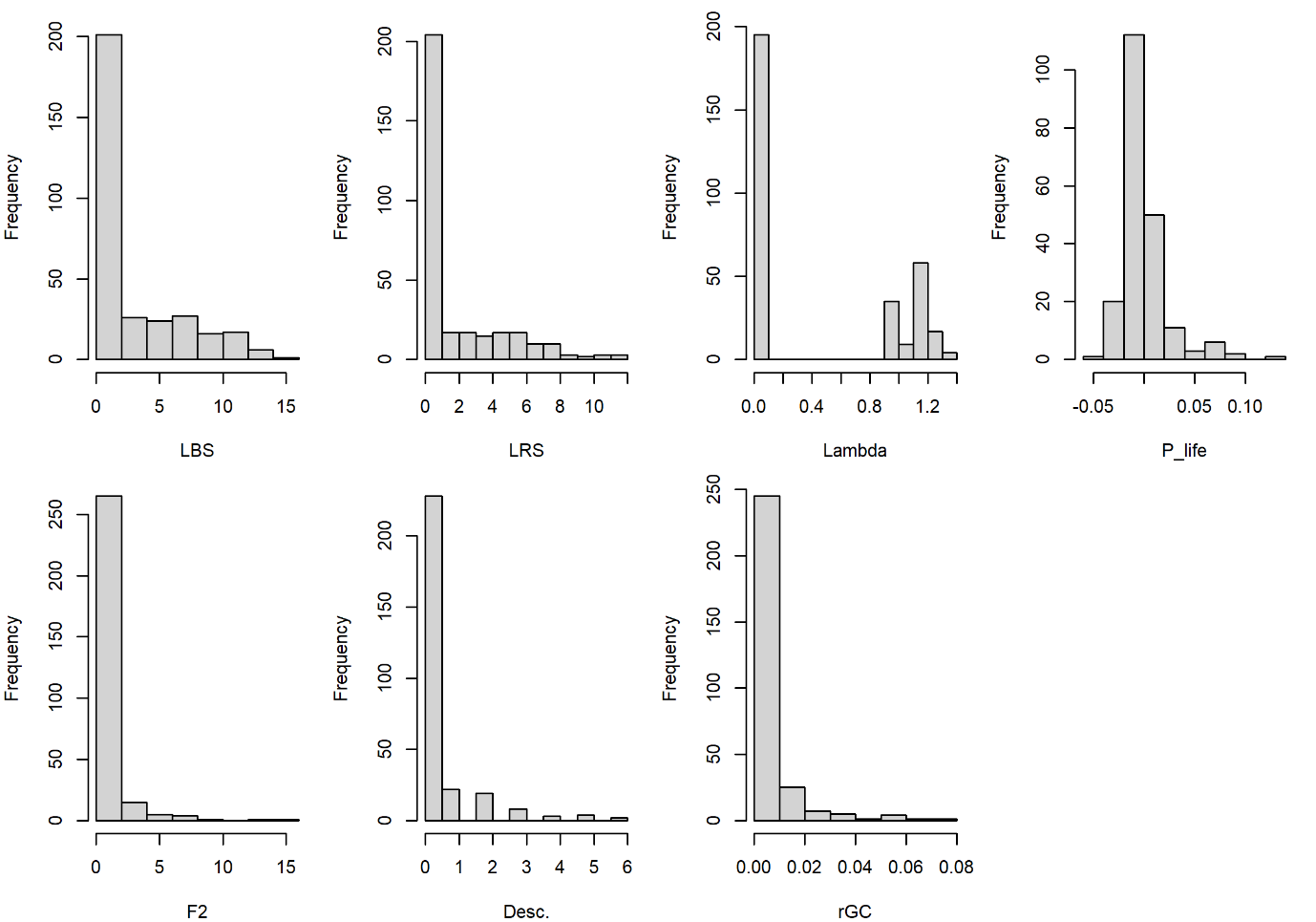


**Figure S9:** Histograms showing the distribution of the seven different proxies calculated on female bighorn sheep from Ram Mountain, Canada, including females that have never breed.

**References**

Aiello, S., Eckstrand, E., Fu, A., Landry, M., & Aboyoun, P. (2016). Machine Learning with R and H2O. *H2O booklet*, *550*.

Bergstra, J., & Bengio, Y. (2012). Random search for hyper-parameter optimization. Journal of machine learning research, 13(2).

Caswell, H. 2001. Matrix population models: construction, analysis, and interpretation (2nd editio.). Sinauer Associates, Sunderland.

Gaillard, J.-M., M. Festa-Bianchet, D. Delorme, and J. T. Jorgenson. 2000. Body mass and individual tness in female ungulates: bigger is not always better. Proceedings of the Royal Society London B 267:471–477.

LeDell, E., Gill, N., Aiello, S., Fu, A., Candel, A., Click, C., Kraljevic, T., Nykodym, T., Aboyoun, P., Kurka, M., & Malohlava, M. (2021). h2o: R Interface for the 'H2O' Scalable Machine Learning Platform. R package version 3.32.1.3. <https://CRAN.R-project.org/package=h2o>

Mcgraw, J. B., and H. Caswell. 1996. Estimation of individual fitness from life-history data. American Naturalist 147:47–64.

Peck, R. (2016, June 6). Hyperparameter Optimization in H2O: Grid Search, Random Search and the Future. https://www.h2o.ai/blog/hyperparameter-optimization-in-h2o-grid-search-random-search-and-the-future/
